# Non-invasive Brain Stimulation, a Tool to Revert Maladaptive Plasticity in Neuropathic Pain

**DOI:** 10.3389/fnhum.2016.00376

**Published:** 2016-07-27

**Authors:** Antonino Naro, Demetrio Milardi, Margherita Russo, Carmen Terranova, Vincenzo Rizzo, Alberto Cacciola, Silvia Marino, Rocco S. Calabro, Angelo Quartarone

**Affiliations:** ^1^IRCCS Centro Neurolesi “Bonino-Pulejo”Messina, Italy; ^2^Department of Biomedical, Dental Sciences and Morphological and Functional Images, University of MessinaMessina, Italy; ^3^Department of Clinical and Experimental Medicine, University of MessinaMessina, Italy

**Keywords:** TMS, neuropathic pain, NIBS, plasticity, tDCS

## Abstract

Neuromodulatory effects of non-invasive brain stimulation (NIBS) have been extensively studied in chronic pain. A hypothetic mechanism of action would be to prevent or revert the ongoing maladaptive plasticity within the pain matrix. In this review, the authors discuss the mechanisms underlying the development of maladaptive plasticity in patients with chronic pain and the putative mechanisms of NIBS in modulating synaptic plasticity in neuropathic pain conditions.

## Introduction

Despite plasticity of the central nervous system is considered a positive adaptive phenomenon related to structural modifications as well as changes in afferent inputs and target outputs, sometimes it may become detrimental causing significant dysfunctions. In this case, functional impairment is the result of maladaptive plasticity (Pascual-Leone et al., 2005).

The best example of maladaptive plasticity in human pathology is focal dystonia where sensory motor plasticity impairment occurs as a consequence of excessive practicing of a stereotyped movement leading to musician’s dystonia or writer’s cramp (Quartarone et al., 2006).

Non-invasive brain stimulation (NIBS) has a therapeutic potential in focal dystonia, as revealed by clinical studies that have demonstrated the efficacious and long-lasting neuromodulatory effects of repetitive transcranial magnetic stimulation (rTMS) at 1 Hz over primary somatosensory area (S1; Havrankova et al., 2010) and rTMS at 0.2 Hz or 1 Hz over the premotor cortex (Murase et al., 2005; Borich et al., 2009).

Chronic pain is another classic example of maladaptive plasticity in neurology and provides the ideal model to discuss the use of NIBS in the prevention of this pathological event.

Therefore, in the present review, we would like to discuss the potential role of NIBS in blocking and possibly reverting maladaptive plasticity, which is associated with several models of chronic pain, such as central post-stroke pain, pain after spinal cord injury or post-surgical pain.

## Maladaptive Plasticity in Chronic Pain

The detection of noxious stimuli (Sherrington, 1906) is a protective process that helps to prevent injury by generating both a reflex withdrawal from the stimulus and a sensation so unpleasant that culminates in complex behavioral strategies to avoid further contact with such noxious stimuli. If stimuli are particularly intense, sensitization of the nociceptive system may lower the threshold for nociception, increasing the amplitude of withdrawal responses to subsequent inputs (Woolf and Salter, 2000). In this sense, nociceptor-induced sensitization of the somatosensory system is a very efficient adaptive plastic mechanism that makes the system hyper alert in conditions in which a risk of further damage is high, for example, immediately after exposure to an intense or damaging stimulus.

In many clinical syndromes, pain is no longer protective. The pain in these situations arises spontaneously, can be elicited by normally innocuous stimuli (allodynia), is exaggerated and prolonged in response to noxious stimuli (hyperalgesia), and spreads beyond the site of injury (secondary hyperalgesia). Overstimulation of nociceptive pathways induced by chronic conditions (such as inflammatory pain, neuropathic pain, or deafferentation syndromes) in predisposed patients (depending on the influence of the individual genotype on the predisposition to pain chronicity and, consequently, the response to treatment; Baron, 2006) may lead to a massive maladaptive re-arrangement in pain-related structures, called central sensitization, which culminates in secondary hyperalgesia and allodynia. When neurons in the dorsal horn of spinal cord are subject to central sensitization, they develop: (i) an increase in spontaneous activity; (ii) a reduction in the threshold for activation by peripheral stimuli; (iii) an increase in response to supra-threshold stimulation; and (iv) an enlargement of their receptive fields (Woolf and King, 1990; Woolf and Salter, 2000; Ji et al., 2003). Central sensitization induces conversion of nociceptive-specific neurons to wide-dynamic-range neurons that now respond to both innocuous and noxious stimuli (Woolf, 1983, 2007).

In this way, spinal dorsal horn neurons undergoing central sensitization become hyper-excitable and hyper-responsive to nociceptive inputs from already sensitized or injured first order neurons. They also show hyper-responsiveness to inputs from other non-sensitized neurons outside the lesioned area (secondary hyperalgesia) and become responsive to non-nociceptive inputs to the nociceptive pathway (allodynia; Woolf, 2011).

At molecular level, central sensitization of pain is characterized by two different phases: (i) the phosphorylation-dependent stage, resulting in rapid changes of glutamate receptors and ion channel properties. This stage is induced with a short latency (seconds) by intense, repeated, or sustained nociceptor inputs and typically lasts from tens of minutes to several hours in the absence of further nociceptor input. (ii) the transcription-dependent stage, where synthesis of new proteins take place for longer-lasting effects. Both these stages depend on N-methyl-D-aspartate (NMDA) receptors and glutamate signaling modifications and contribute to the induction and maintenance of acute activity-dependent central sensitization (Woolf and Thompson, 1991). Multiple triggers can contribute to the establishment of this process, such as substance P, Calcitonin Gene Related Peptide (CGRP), bradykinin, Brain-Derived Neurotrophic Factor (BDNF), and nitric oxide (Latremoliere and Woolf, 2009). Indeed, these different triggers are released or induced in response to nociceptor activity, and each trigger can initiate the activation of multiple intracellular signaling pathways that lead to a hyperexcitability in dorsal horn neurons. The elevation in intracellular Ca^2+^ has a key role since it activates multiple Ca^2+^-dependent kinases acting on receptors and ion channels, which increases synaptic efficacy.

Finally, glutamate receptor phosphorylation during central sensitization increases the activity/density of NMDA receptors, leading to an increase in membrane excitability, a facilitation of synaptic strength, a decrease in inhibitory influences in dorsal horn neurons, and the strengthening of nociceptive transmission at the dorsal horn. The role of glutamate in central sensitization is suggested by animal studies that have revealed that NMDA receptor blockade by microinjection of 2-amino-5-phosphonopentanoate in the rostral ventromedial medulla (RVM) attenuated signs of central sensitization (Coutinho et al., 1998; Urban et al., 1999). Similarly, microinjection of MK-801 (a NMDA receptor antagonist) within the thalamus reduces signs of central sensitization (Kawamura et al., 2010; Kaneko et al., 2011). The NIBS-induced plasticity modulation is achieved though several mechanisms, including changes in threshold, in kinetics and trafficking to the membrane of glutamate receptors, increase in inward currents and reduction in outward currents of ion channels, and reduction in inhibitory neurotransmission. Altogether, such mechanisms may lead to changes in the excitability of nociceptive neurons (Carvalho et al., 2000; Fang et al., 2003).

On the other hand, transcription-dependent changes are required for longer-lasting effects; these do not occur only in response to nociceptor activity but also as a consequence of peripheral inflammation and nerve injury (see below). In this stage, different mechanism of synaptic plasticity with some resemblance to long-term potentiation (LTP) and long-term depression (LTD) phenomena occur in central nervous system, thus activating either active synapses (homosynaptic potentiation) or non-activated synapses (heterosynaptic potentiation). The main mediators of these mechanism are thought to be the metabotropic glutamate receptors and the nitroxide (Fagni et al., 2000).

Even though the role of neural circuit remodeling and structural synaptic plasticity in the “pain matrix” in chronic pain has been thought as a secondary epiphenomenon to altered nociceptive signaling in the spinal cord, brain imaging studies on human patients and animal models have suggested the possibility that structural plastic changes in cortical neural circuits may actively contribute to the development of chronic pain symptoms (Kim and Kim, 2016). Indeed, activity-dependent central sensitization is basically an adaptive mechanism, since it prevents, e.g., the use of an injured body part. Nonetheless, central sensitization is pathological when tissue damage persists or if it becomes autonomous and it is maintained in absence of real signaling (Koltzenburg et al., 1992b).

At central level, the abovementioned plastic changes indeed occur in at least six supra-spinal structures of the pain matrix, including the primary somatosensory cortex (S1), secondary somatosensory cortex (S2), anterior cingulate cortex (ACC), insular cortex, and thalamus, which are involved in the phenomena of central sensitization (Urban and Gebhart, 1999; Zhuo, 2007). In addition, neuroimaging studies of induced secondary hyperalgesia have shown significant activations in the prefrontal cortex, periaqueductal gray (PAG), nucleus cuneiformis, superior colliculi, cerebellum and somatosensory and parietal associative cortices (Iadarola et al., 1998; Baron et al., 1999; Witting et al., 2001; Maihöfner et al., 2004; Zambreanu et al., 2005; Lee et al., 2008; Seifert et al., 2009). On the other hand, pathological and experimentally induced allodynia appear to be associated with enhanced activity of ACC, thalamus, RVM, PAG, insula, orbitofrontal cortex, dorsolateral prefrontal cortices (DLPFCs), putamen, somatosensory cortex, and dorsomedial midbrain. These supra-spinal structures may exert facilitatory pain mechanisms that have been implicated in the generation and maintenance of central sensitization and, possibly, the establishment of chronic pain (Lorenz et al., 2002, 2003; Becerra et al., 2006; Mainero et al., 2007; Seifert and Maihöfner, 2007; Geha et al., 2008).

By a molecular point of view, the NMDA-mediated mechanisms of central sensitization also contributes to the longer-lasting and sometimes persistent pain hypersensitivity (Latremoliere and Woolf, 2009). With regard to neuropathic pain, damaged and non-damaged Adelta- and C-fiber generate spontaneous action potentials after a peripheral nerve injury (ectopic input; Devor and Seltzer, 1999; Djouhri et al., 2006). Such activity in C- and also Adelta-fiber can initiate and maintain activity-dependent central sensitization in the dorsal horn (Koltzenburg et al., 1992a; Devor, 2009). Injured and also non-injured sensory neurons in the dorsal root ganglion exhibit massive changes in transcription, thus altering their membrane properties, growth, and transmitter functions (Xiao et al., 2002). Affected fibers express new transmitters and neuromodulators, including substance P, BDNF, and a cofactor for nitroxide synthase (namely, synthetic enzymes for tetrahydrobiopterin). On the other hand, the stimulation of non-nociceptive fibers triggers the release of factors that can further drive central sensitization (Xiao et al., 2002). The release of these mediators induces a substantial disinhibition in the dorsal horn with loss of Gamma-Aminobutyric-Acid(GABA)ergic and glycinergic inhibitory currents leading to a NMDA-dependent excitotoxicity neuronal death (Moore et al., 2002; Scholz et al., 2005). In addition, there is also an increase in descending excitatory controls from the RVM in the brainstem after peripheral nerve injury, as well as a reduction of descending inhibitory controls (Vera-Portocarrero et al., 2006).

Of note, there are also structural changes as a consequence of the molecular processes described above, which consist in a trans-ganglionic degeneration of C-fiber terminals in lamina II. This degeneration determines the myelinated Abeta-fibers sprouting from laminae III-IV into laminae I-II and making contact with nociceptive-specific neurons (Woolf et al., 1992, 1995). Finally, astrocytes become hyper-active after nerve injury and may play a role in the maintenance of neuropathic pain hypersensitivity (Zhuang et al., 2005).

It is likely that chronic pain, regardless of the etiology (inflammatory or neuropathic) and pain model, may trigger various forms of maladaptive structural plasticity at cortical and sub-cortical level, which in turn could be directly or indirectly involved in the development of sensory, emotional and cognitive symptoms of chronic pain. Since it is well known that structural plasticity of neuronal connections in the brain occurs after a period of several weeks or months after the functional changes, it is mandatory in the future, to intervene as soon as possible before these permanent changes may take place. In this perspective, the use of NIBS in the transition from acute to chronic pain should be explored in the near future to optimize a time window for new efficient therapeutic strategies (Andrade et al., 2013).

## General Overview on Non-Invasive Brain Stimulation and Cortical Plasticity: TMS and Transcranial Direct Current Stimulation (tDCS)

TMS and tDCS are methods to painlessly stimulate the cerebral cortex through the intact skull, and can be used to induce long-term effects in several cortical areas.

TMS was conceived as a method to investigate the integrity of the corticospinal outflow from cerebral motor cortex to the spinal cord (Rothwell, 1997). Indeed, TMS pulses readily penetrate the skull and carry an electric stimulating current into the cortex near the surface, thus activating the axons of interneurons of layers II and III that synapse onto the pyramidal neurons of layers V. In this way, the size of the response produced by a given stimulus is sensitive to the excitability of synaptic connections within the cortex, giving an indirect measure of the excitability of intrinsic cortical circuits within the conscious brain. This provides a reliable indicator of any changes produced by neural plasticity within the motor cortex. In addition, when probing motor cortex excitability with single pulses, TMS can also produce long-term changes in excitability if the TMS pulses are applied repetitively (Siebner and Rothwell, 2003). In general, low-frequency stimulation (1 Hz or below) depresses cortical excitability, whereas high-frequency application (5 Hz) increases cortical excitability (Quartarone et al., 2005a). Although the duration of the effects of brief rTMS is short-lasting, longer-lasting after-effects can be achieved by using protocols that include longer periods of stimulation or multiple sessions of rTMS (Quartarone et al., 2006).

Most researchers believe that the long-lasting therapeutic effects of rTMS and the effects of magnetic stimulation on the processes described above are related to two phenomena: LTP and LTD (Ziemann, 2004). The possibility that rTMS induces changes in brain excitability that outlast the stimulation period has prompted its use for therapeutic purposes. The long lasting effects are probably mediated through NMDA synaptic plasticity. Indeed, it has been demonstrated that the long lasting after effects of continuous and intermittent theta burst stimulation on the M1 of healthy volunteers are abolished by using memantine, an NMDA-receptor antagonist (Huang et al., 2008). According to the classical model of induction of LTP- and LTD-like effects, postsynaptic NMDA receptors induce Ca^2+^ influx into neurons. This ionic shift triggers a series of reactions that prompt long-term changes in synaptic strength (Malenka and Bear, 2004). An important upstream regulator of NMDA synaptic plasticity is the BDNF- Tropomyosin receptor kinase-B (TrkB) system. Indeed, we showed that a 5-day rTMS stimulation enhanced BDNF binding affinity for TrkB, BDNF-TrkB signaling, and NMDA receptor-TrkB interaction in rat prefrontal cortex (Wang et al., 2011). Interestingly, in the same study, we showed that the same protocol could induce an increased BDNF binding affinity for TrkB and enhanced BDNF-TrkB signaling in rats and humans peripheral lymphocytes (Wang et al., 2011). These results suggest that the long lasting excitatory effects of rTMS are at least in part mediated through an upstream regulation of glutamatergic NMDA interaction.

Another important issue about the mechanism of action of rTMS at a system level is that the effects of rTMS are not only restricted to the stimulated region but tend to spread over distant interconnected cortical, subcortical, and spinal structures (Kobayashi and Pascual-Leone, 2003). This possibility opens a window to reach subcortical structures of the pain matrix that are involved in the mechanism of central sensitization. Indeed, neuroimaging studies have revealed that rTMS applied over the primary motor cortex (M1) can modulate the activity in cortical and subcortical regions such as M1, premotor cortex supplementary motor area, thalamus, ACC, somatosensory cortex, insula, red nucleus, and cerebellum (Fox et al., 1997; Siebner et al., 2000; Baudewig et al., 2001; Lee et al., 2003; Okabe et al., 2003; Speer et al., 2003; Takano et al., 2004; Rounis et al., 2005; Gaynor et al., 2008; Cárdenas-Morales et al., 2011). Similarly, DLPFC, ACC, somatosensory cortex, basal ganglia, thalamus, insula, cerebellum and parahippocampus were the main targeted areas when rTMS was applied over DLPFC (Zheng, 2000; Paus et al., 2001; Loo et al., 2003; Michael et al., 2003; Ferrarelli et al., 2004).

tDCS does not induce any action potential, in contrast to other NIBS techniques. It instead modulates membrane excitability by the application of weak electrical currents through two oppositely charged electrodes. The amount of polarization is small, but it can bias the membrane potential of cells, changing the threshold for synaptic activation. When a positively charged electrode (anode) is applied to the surface of the scalp, a fraction of the current is thought to enter the brain and to polarize neurons in proximity of the electrode, thus increasing neuronal firing. Conversely, a negatively charged electrode (cathode) decreases cortical excitability and induces neuronal hyperpolarization (Nitsche and Paulus, 2000; Quartarone et al., 2006).

Application of a small current (1–2 mA), using two electrodes on the scalp for 5–10 min, changes cortical excitability up to 30–60 min afterward (Nitsche and Paulus, 2000). Animal experiments show that this leads to changes in firing rates of neurons while the stimulus is applied, and it is thought that this causes long-term effects on excitability that outlast the stimulation (Fritsch et al., 2010). Similar to the effects of rTMS, after-effects of tDCS are abolished by NMDA receptor antagonists and, hence, are likely to reflect changes in synaptic effectiveness (Nitsche et al., 2003). In addition to NMDA receptors, it is also possible that dopamine and GABA receptors are involved in tDCS-mediated neuroplasticity. Indeed the administration of sulpiride (a D2 receptor antagonist) abolishes tDCS after-effects in normal humans (Nitsche et al., 2006). In addition, lorazepam enhances and prolongs the plastic effects of anodal tDCS (Nitsche et al., 2004). Finally, the effects of tDCS can also be non-synaptic, possibly involving transient changes in the density of protein channels localized below the stimulating electrode or alterations on cAMP and calcium levels (Nitsche et al., 2008). Indeed, the tDCS-induced constant electric field can locally change ionic concentrations, induce a migration of transmembrane proteins (similarly to gel electrophoresis), thus causing steric and conformational changes, and locally alter the tissue acid–base balance (Ardolino et al., 2005). The latter may mainly affect NMDA signaling (Tang et al., 1990).

## Putative Mechanisms of TMS in Pain Treatment

Best practices for neurostimulation on neuropathic pain have been standardized and are available in the European Federation of Neurological Societies for neurostimulation therapy for neuropathic pain (Cruccu et al., 2007). Nonetheless, it is difficult to determine which specific parameters are best for clinical use, since the TMS treatment parameters vary among the published studies. Effectiveness of rTMS depends on the type of neuropathic pain (Lefaucheur, 2006; Leung et al., 2013), although many types of intractable chronic pain have been treated with NIBS. On note, before rTMS can be applied in a patient, it is necessary to accurately determine timing, amount, and duration for each stimulation session, thereby ensuring the optimal duration of effect. Significant results have been reported when employing rTMS at 20 Hz (Fricová et al., 2009; Leung et al., 2013). Nonetheless, rTMS has also been tested at low-frequency stimulation (1 Hz), thus reducing the activity of excitatory circuits in the human motor cortex. However, the best frequency of stimulation for the most effective pain treatment has not yet been resolved. The most commonly targeted area is represented by the M1 contralateral to the position corresponding to the somatotopic location of the pain source; the DLPFC is also of interest, since it seems to have a substantial influence on neuronal circuits involved in the processing of cognitive and emotional aspects of pain (Rokyta and Fricová, 2012).

Beyond frequency and protocol duration, the orientation of the figure-of-eight–shaped coil used to perform the stimulation can influence the nature of the descending volleys elicited by the TMS itself. It is well known that the best analgesic effect is obtained using an antero-posterior orientation (André-Obadia et al., 2008). Taking into account the effects of magnetic field orientation on cortical fibers, pain relief after stimulation of M1 is thought to be produced by activating fibers running superficially within the precentral gyrus, parallel to the convexity of the cortical surface. This pattern of activation is similar to that produced by cathodal epidural motor cortex stimulation (EMCS) at the crown of the precentral gyrus.

Another important issue when designing a NIBS protocol for pain treatment is the timing of rTMS application. It is generally thought that rTMS should be applied as soon as possible in case of intractable pain (Treister et al., 2013).

There are many uncertainties regarding the mechanism of pain relief induced by TMS and the nature and connections of the TMS-activated neuronal circuits (Nguyen et al., 2011). It is thought that NIBS may target the ‘top–down’ regulatory system controlling anti-nociception. TMS may induce a variety of changes concerning LTD and LTP mechanisms, activation of feedback loops, and changes in neuronal excitability. In fact, neurostimulation can activate axons more easily than cell bodies (Nowak and Bullier, 1998) and, therefore, the mechanisms of action of neurostimulation must be modeled in terms of neural circuits rather than local brain activity changes. Axons recruited by cortical stimulation can be short fibers of intracortical interneurons of layers II and III and afferent or efferent fibers connected with distant structures. Altogether, these changes may decrease sensory pain threshold and inhibits the transmission of sensory information in the spinothalamic tract, depending on the stimulation duration and frequency of each treatment (Lefaucheur et al., 2004; Lefaucheur, 2008).

Of note, the fact that motor but not sensory cortex stimulation relieves pain is not fully understood. Since TMS only directly affects the superficial cortex, the currents rapidly dissipate, the triggered action potentials propagate to distributed neural networks, and M1 projections directly reach pain-modulating structures (including medial thalamus, anterior cingulate/orbitofrontal cortices, and PAG), it is possible that parallel fibers within motor areas may be more suitable than the sensory ones to be targeted by TMS (Irlbacher et al., 2006; Wasserman et al., 2008; Mylius et al., 2012; Peterchev et al., 2012). Indeed, experimental evidence suggests that either epidural stimulation or NIBS may act through an antidromic modulation of the thalamo-cortical pathways (Tsubokawa et al., 1991), thus confirming the important role of the connections between afferent fibers from thalamic nuclei and pyramidal cells concerning nociception control (Villanueva and Fields, 2004). In keeping with this notion, recent studies confirmed that the integrity of the thalamo-cortical tract is required to mediate the anti-nociceptive effects of high-frequency rTMS of M1 (Ohn et al., 2012). In addition, there is evidence suggesting that rTMS may exert a descending modulation within the brainstem, triggered by the cortico-thalamic output (Lefaucheur et al., 2004).

Finally, it should be considered that rTMS of M1 also can act on structures involved in the affective, cognitive, and emotional aspects of pain, such as the cingulate, prefrontal, and orbitofrontal cortices involving opioidergic mechanisms (Tamura et al., 2004). In line with this view, an elevation of serum beta-endorphin concentration was found in patients with phantom limb pain successfully treated by high-frequency rTMS of M1 (Ahmed et al., 2011). Last, naloxone (an opioid receptor antagonist) significantly reduces the analgesic effect of high-frequency rTMS on either M1 or left DLPF in normal volunteers (de Andrade et al., 2011; Taylor et al., 2012). Regarding the neurotransmitters, the mechanisms of action of motor cortex stimulation could also involve inhibitory GABA transmission. This is suggested by some data reporting that intracortical inhibition, a TMS marker of GABA_A_ transmission in the motor cortex, is reduced in the hemisphere contralateral to neuropathic pain. High-frequency rTMS of M1 can restore intracortical inhibition in correlation with the amount of induced pain relief in patients with neuropathic pain (Lefaucheur et al., 2006, 2012; Fierro et al., 2010; Mhalla et al., 2011).

## Putative Mechanisms of tDCS in Pain Treatment

As compared to TMS, tDCS after-effects are less well characterized (Ngernyam et al., 2013). There is growing evidence confirming the effectiveness of tDCS in treating different types of neuropathic pain (Knotkova et al., 2013), including refractory orofacial pain, fibromyalgia, phantom pain, and back pain (Rokyta et al., 2012; Bolognini et al., 2013; Zhang et al., 2013). Several papers have used different sites of stimulation, including the DLPFC and M1 (Fregni et al., 2006a,b; O’Connell et al., 2013), intensity of stimulation (1–2 mA), time (from 10 up to 30 min; Boggio et al., 2009) and duration of application (i.e., number of sessions per week; Soler et al., 2010).

The mechanism of action of tDCS differs from that of rTMS or epidural motor cortex stimulation, since tDCS-induced current intensity is not high enough to generate action potentials into the brain by itself alone (Lefaucheur, 2016). As outlined above, tDCS increases or decreases the value of axon membrane potential (depolarization or hyperpolarization), according to the polarity (anodal or cathodal) of the stimulation. However, tDCS may exert local and remote effects that, like rTMS, extend well beyond the time of stimulation, reversibly, painlessly, and safely (Nitsche and Paulus, 2001).

Similarly to EMCS and rTMS, the analgesic effects of tDCS may result from the modulation of distant neural structures involved in sensory-discriminative, cognitive, or emotional aspect of chronic pain (Yoon et al., 2014). Indeed, tDCS has preferential analgesic efficacy when the motor cortex receives anodal stimulation, whereas EMCS-induced analgesia is mediated by the placement of cathode over M1 (Holsheimer et al., 2007b; Foerster et al., 2015). This is supported by a recent study showing decreased levels of glutamate in the ACC and thalamus and increased levels of N-acetyl-aspartate and GABA in the posterior and anterior insula after anodal tDCS delivered over the left M1 in patients with non-neuropathic pain (DosSantos et al., 2012). In addition, similarly to rTMS, tDCS may also target the opioid system. In particular, the posterior thalamus was activated by anodal tDCS of M1 in a patient with trigeminal neuropathic pain (Holsheimer et al., 2007a).

## Expert Commentary and Future Perspectives

In line with the current lines of research, we hypothesize that NIBS over M1 could exert its modulation of descending facilitatory pathways and the subsequent disruption of ongoing plastic changes in cortical and sub-cortical structures of the pain matrix, before they consolidate in maladaptive structural phenomena.

Considering that central sensitization is a mechanism mediated by NMDA related synaptic plasticity, it is tempting to consider the possibility of using rTMS at early stages to shift the threshold of plasticity and to trigger homeostatic mechanisms that could reset abnormal plasticity and may prevent the development of maladaptive plasticity phenomena.

In line with this hypothesis, one opportunity of manipulating the abnormal plasticity in acute pain would be to prime the effects of rTMS. Indeed, preconditioning M1 using tDCS prior to 1 Hz rTMS of M1 effectively modulated experimental thermal pain thresholds. In addition, the direction of pain threshold modulation after 1 Hz rTMS depended on the polarity of tDCS priming. For the cathodal (inhibitory) tDCS before 1 Hz rTMS, heat and cold pain thresholds significantly increased. Consistently with the concept that pre-conditioning with tDCS controls the direction of the effect of subsequent rTMS, pain threshold decrease was observed after the anodal (excitatory) tDCS before 1 Hz rTMS (Moloney and Witney, 2013).

Further studies are needed to provide direct evidence of the efficacy of NIBS to prevent the development of maladaptive plasticity at an early stage, using the prime technique. In particular, it would be important to evaluate the homeostatic control of plasticity in patients with neuropathic pain, especially in the acute phase, in order to better define the priming protocol of stimulation.

It is interesting to note that patients suffering from migraine have an alteration of the homeostatic regulation plasticity within the motor cortex between the attacks (Antal et al., 2008), similarly to patients with focal dystonia, another condition characterized by maladaptive plasticity (Quartarone et al., 2005b; Kang et al., 2011).

Finally, since there are no reliable serum biological markers that can assess neuroplasticity, it will be useful to validate surrogate outcomes for neuroplasticity using TMS, high-density electroencephalography, and neuroimaging methods (including tractography), in the attempt to better correlate functional and structural maladaptive plastic changes with clinical outcomes.

## Author Contributions

AN: work conception and design, work revision, final approval, global agreement. DM: data acquisition, data analysis, data interpretation, work revision, final approval, global agreement. MR: work conception and design, work revision, final approval, global agreement. CT: data acquisition, data analysis, data interpretation, drafting the work, final approval, global agreement. VR: work conception and design, drafting the work, final approval, global agreement. AC: data acquisition, data analysis, data interpretation, drafting the work, final approval, global agreement. SM: work conception and design, work revision, final approval, global agreement. RC: data acquisition, data analysis, data interpretation, work revision, final approval, global agreement. AQ: work conception and design, drafting the work, final approval, global agreement.

## Conflict of Interest Statement

The authors declare that the research was conducted in the absence of any commercial or financial relationships that could be construed as a potential conflict of interest.
